# Stability of Nonlinear Dirichlet BVPs Governed by Fractional Laplacian

**DOI:** 10.1155/2014/920537

**Published:** 2014-03-02

**Authors:** Dorota Bors

**Affiliations:** Faculty of Mathematics and Computer Science, University of Lodz, Banacha 22, 90-238 Lodz, Poland

## Abstract

We consider a class of partial differential equations with the fractional Laplacian and the homogeneous Dirichlet boundary data. Some sufficient condition under which the solutions of the equations considered depend continuously on parameters is stated. The application of the results to some optimal control problem is presented. The methods applied in the paper make use of the variational structure of the problem.

## 1. Introduction

Consider the following fractional partial differential equation with some variable distributed parameters of the form

(1) $$\begin{array}{c} {\left(-\Delta \right)}^{\alpha /2}u\left(x\right)+\varphi \left(x,u\left(x\right),\omega \left(x\right)\right)=0\;\text{for}\;x\in \Omega \subset {\mathbb{R}}^{n}, \end{array}$$

(2) $$\begin{array}{c} u\left(x\right)=0\;\text{for}\;x\in {\mathbb{R}}^{n}\setminus \Omega , \end{array}$$

where *n* ≥ 2, *Ω* is a bounded domain with a Lipschitzian boundary ∂*Ω*, and *u* ∈ *H*_0_^*α*/2^(*Ω*, ℝ) with *α* ∈ (0,2). We shall assume that the distributed parameter *ω* belongs to the space *L* ^*p*^(*Ω*, ℝ^*m*^) for some suitably chosen *p* > 1 and *m* ≥ 1.

The equation under consideration is the generalization of the nonlinear Poisson equation involving the Brownian diffusion expressed by the local Laplace operator fully analyzed in [1–3]. We extend our considerations to cover also the case of the nonlocal, fractional Laplace operator being the infinitesimal generator of Lévy processes; see, for instance, [4–7], allowing, contrary to the continuous Brownian motion, for jumps. We prove the analogous stability results as for the Brownian motion with the Laplace operator involved obtained in [1–3].

The problems with the fractional Laplacian attracted in recent years a lot of attention as they naturally arise in various areas of applications to mention only [5–11] and references therein. They appear in probabilistic framework as well as in mathematical finance as infinitesimal generators of stable Lévy processes [4–7]. Moreover one can find the problems involving fractional Laplacian in mechanics and in elastostatics, for example, in Signorini obstacle problem originating from linear elasticity [12–14] as well as in fluid mechanics and in hydrodynamics—appearing in quasi-geostrophic fractional Navier-Stokes equation [15] and describing some porous media flows in the hydrodynamic model like in [11]. The author considered also global solvability of Hammerstein equations derived from BVPs involving fractional Laplacian in recent paper [16].

In the theory of boundary value problems (BVPs) and its applications one considers, first of all, the problem of the existence of a solution, next the question of its stability, uniqueness, and smoothness, and finally the issue of asymptotic analysis. One can say that a given problem is well posed if the problem possesses at least one solution or, more generally, one obtains the set of solutions, which continuously changes along with the change of variable parameters of the system which we call stability. Otherwise we refer to the problem as to ill-posed one. The requirement of stability is necessary if the mathematical formulation is to describe observable natural phenomena, which by its very nature cannot possibly be conceived as rigidly fixed: even the mere process of measuring them involves small errors as was noted by Courant and Hilbert in [17]. The theory of ill-posed problems pays most attention to the requirement of the stability of the boundary value problems.

In this paper we formulate some sufficient condition under which the boundary value problem considered here possesses at least one solution which continuously depends on distributed parameters. The problem of controllability of the related evolution equations driven by the anomalous diffusion governed by the fractional Laplacian was considered, for example, in [18].

The paper is organized as follows. In Section 2 we formulate the problem and list the assumptions appearing throughout the paper. In Section 3, using some variational methods we prove that boundary value problem (1)-(2) is stable with respect to the norm topology in the space of distributed parameters *L* ^*p*^(*Ω*, ℝ^*m*^) and the norm topology in the fractional Sobolev space of solutions *H* ^*α*/2^(*Ω*, ℝ). We can formulate the main result of Section 3 as follows: if *ω*_*k*_ → *ω*_0_ in *L* ^*p*^(*Ω*, ℝ^*m*^), then *u*_*k*_ → *u*_0_ in *H* ^*α*/2^(*Ω*, ℝ) where *u*_*k*_ is the solution of the boundary value problem (1)-(2) with fixed *ω* = *ω*_*k*_, *k* ∈ *ℕ*_0_ under suitable conditions imposed on *φ*. In the case when (1) is linear with respect to *ω*, we can relax the topology in the space *L* ^*p*^(*Ω*, ℝ^*m*^). In short, in Section 4, we prove that *u*_*k*_ → *u*_0_ in *H* ^*α*/2^(*Ω*, ℝ) provided that *ω*_*k*_⇀*ω*_0_ weakly in *L* ^*p*^(*Ω*, ℝ^*m*^). In the next section, we present a theorem on the existence of an optimal solution to some control problem with the integral cost functional. The proof of this theorem relies in essential way on the continuous dependence results. In the final part of the paper we give a short survey of the results related to the stability of the initial and boundary value problems for the second-order partial differential systems with parameters.

## 2. Formulation of the Problem, Introduction of the Fractional Laplacian, and Basic Assumptions

For the definition of the fractional Laplacian one can see [19–25]. In particular, we denote by (*u*_*j*_, *ρ*_*j*_) for *j* ∈ *ℕ* the system of the eigenfunctions and eigenvalues for the Laplace operator −Δ on *Ω* with the homogeneous Dirichlet condition on ∂*Ω*. Moreover, by *H*_0_^*α*/2^(*Ω*, ℝ), let us denote the Sobolev space of functions *u* = *u*(*x*) defined on a bounded, smooth domain *Ω* ⊂ ℝ^*n*^, *n* ≥ 2, such that *u* = ∑_*j*=1_^*∞*^ *a*_*j*_ *u*_*j*_ and ∑_*j*=1_^*∞*^ *a*_*j*_^2^ *ρ*_*j*_^*α*/2^ < *∞*, with the norm in *H*_0_^*α*/2^(*Ω*, ℝ) with *α* ∈ (0,2) defined by the equivalent formulas

(3) $$\begin{array}{c} {||u||}_{H_{0}^{\alpha /2}}^{2}=\sum_{j=1}^{\infty }a_{j}^{2}\rho_{j}^{\alpha /2}={||{\left(-\Delta \right)}^{\alpha /4}u||}_{L^{2}}^{2}={||{\left(-\Delta \right)}^{\alpha /2}u||}_{H^{-\alpha /2}}^{2}, \end{array}$$

see, for example, [20, 23] and for the last equality, see, for example, [19]. The fractional Laplacian acts on *u* = ∑_*j*=1_^*∞*^ *a*_*j*_ *u*_*j*_ as

(4) $$\begin{array}{c} {\left(-\Delta \right)}^{\alpha /2}u=\sum_{j=1}^{\infty }a_{j}\rho_{j}^{\alpha /2}u_{j}. \end{array}$$

The fractional Sobolev spaces are also referred to as Gagliardo or Slobodeckij spaces. One can give yet another definition of *H* ^*α*/2^(*Ω*, ℝ) as follows:

(5) Hα/2(Ω,ℝ) ={u∈L2(Ω,ℝ):|u(x)−u(y)||x−y|(n+α)/2∈L2(Ω×Ω,ℝ)}

with the norm

(6) $$\begin{array}{c} {||u||}_{H^{\alpha /2}}^{2}=\int_{\Omega }^{}{\left|u\left(x\right)\right|}^{2}dx+\iint_{\Omega \times \Omega }^{}\frac{{\left|u\left(x\right)-u\left(y\right)\right|}^{2}}{{\left|x-y\right|}^{n+\alpha }}dx\;dy. \end{array}$$

For the definition of the fractional Laplacian operator involving singular integrals consistent with ours when *u* is extended by 0 outside *Ω*, we refer the readers to [23], where one can find the following lemma.

**Lemma 1**

Let *α* ∈ (0,2) and let (−Δ)^*α*/2^ be the fractional Laplacian operator of the form

(7) $$\begin{array}{c} {\left(-\Delta \right)}^{\alpha /2}u\left(x\right)=C\left(n,\alpha \right)\underset{ɛ\to 0^{+}}{\lim}\int_{{\mathbb{R}}^{n}\setminus B\left(x,ɛ\right)}^{}\frac{u\left(x\right)-u\left(y\right)}{{\left|x-y\right|}^{n+\alpha }}dy, \end{array}$$

where *C*(*n*, *α*) = *π* ^−(*α*+*n*/2)^(Γ((*n* + *α*)/2)/Γ(−*α*/2)). Then for any *u* from the Schwartz space of rapidly decaying *C* ^*∞*^ functions in ℝ^*n*^ we have

(8) $$\begin{array}{c} {\left(-\Delta \right)}^{\alpha /2}u\left(x\right)=-\frac{1}{2}\int_{{\mathbb{R}}^{n}}^{}\frac{u\left(x+y\right)+u\left(x-y\right)-2u\left(x\right)}{{\left|x-y\right|}^{n+\alpha }}dy \end{array}$$

for all *x* ∈ ℝ^*n*^ (cf. [23, Lemma 3.5]).

Throughout the paper, we shall assume that *Ω* satisfies any condition which guarantees a compact embedding of *H*_0_^*α*/2^(*Ω*, ℝ) into *L* ^*s*^(*Ω*, ℝ) with *s* ∈ (1, 2_α_*) where 2_α_* = 2*n*/(*n* − *α*) if *n* ≥ 2, for example, ∂*Ω* may be Lipschitzian; that is, ∂*Ω* ∈ *C* ^0,1^ (cf. [26] for the definition of *C* ^0,1^). For ∂*Ω* ∈ *C* ^0,1^ it is possible to extend *u* by 0 outside *Ω* and stay in the same space; see [23, Theorem 5.4].

Further, in this paper we shall use the primitive *ϕ* of the mapping *φ* : *Ω* × ℝ × ℝ^*m*^ → ℝ, implying *φ* to be defined as the derivative with respect to *u* variable of a function *ϕ* : *Ω* × ℝ × ℝ^*m*^ → ℝ; that is

(9) $$\begin{array}{c} \varphi \left(x,u,\omega \right)=\phi_{u}\left(x,u,\omega \right), \end{array}$$

where *x* ∈ *Ω* a.e., *u* ∈ ℝ, and *ω* ∈ ℝ^*m*^.

In this case boundary value problem (1)-(2) may be written in the form suitable for variational analysis

(10) $$\begin{array}{c} {\left(-\Delta \right)}^{\alpha /2}u\left(x\right)+\phi_{u}\left(x,u\left(x\right),\omega \left(x\right)\right)=0\;\text{for }x\in \Omega \subset {\mathbb{R}}^{n} \end{array}$$

(11) $$\begin{array}{c} u\left(x\right)=0\;\text{for }x\in {\mathbb{R}}^{n}\setminus \Omega , \end{array}$$

where *ω* ∈ *L* ^*p*^(*Ω*, ℝ^*m*^), *p* > 1 and *m* ≥ 1. It is easily seen that (10)-(11) represent the Euler-Lagrange equation for the following functional of action:

(12) $$\begin{array}{c} F_{\omega }\left(u\right)=\int_{\Omega }^{}\left[\frac{1}{2}{\left|{\left(-\Delta \right)}^{\alpha /4}u\left(x\right)\right|}^{2}+\phi \left(x,u\left(x\right),\omega \left(x\right)\right)\right]dx, \end{array}$$

where *u* ∈ *H*_0_^*α*/2^(*Ω*, ℝ) and *ω* ∈ *L* ^*p*^(*Ω*, ℝ^*m*^). It should be underlined that the solutions of Euler-Lagrange equation (10)-(11) are meant in the weak sense; that is, for any *v* ∈ *H*_0_^*α*/2^(*Ω*, ℝ) the following equality holds:

(13) ∫Ω(−Δ)α/4u(x)(−Δ)α/4v(x)dx  +∫Ωφ(x,u(x),ω(x))v(x)dx=0.

To obtain the existence of the weak solutions of the boundary value problem with fractional Laplacian (10)-(11) in the fractional Sobolev space *H*_0_^*α*/2^(*Ω*, ℝ) and the continuous dependence of solutions on distributed parameters we shall impose on *ϕ* the following conditions.

(A1) regularity: the functions *ϕ* and *ϕ*_*u*_ are measurable with respect to *x* for any (*u*, *ω*) ∈ ℝ × ℝ^*m*^ and continuous with respect to (*u*, *ω*) ∈ ℝ × ℝ^*m*^ for a.e. *x* ∈ *Ω*.

(A2) growth: for *p* ∈ (1, *∞*), there exists a constant *c* > 0 such that

(14) $$\begin{array}{c} \left|\phi \left(x,u,\omega \right)\right|\leq c\left(1+{\left|u\right|}^{s}+{\left|\omega \right|}^{p}\right),\\ \left|\phi_{u}\left(x,u,\omega \right)\right|\leq c\left(1+{\left|u\right|}^{s-1}+{\left|\omega \right|}^{p-p/s}\right), \end{array}$$

for a.e. *x* ∈ *Ω*, *ω* ∈ ℝ^*m*^ and *u* ∈ ℝ, where *s* ∈ (1, 2_α_*) where 2_α_* = 2*n*/(*n* − *α*); for *p* = *∞* and any bounded set *W* ⊂ ℝ^*m*^ there exists a constant *c* > 0 such that

(15) $$\begin{array}{c} \left|\phi \left(x,u,\omega \right)\right|\leq c\left(1+{\left|u\right|}^{s}\right),\\ \left|\phi_{u}\left(x,u,\omega \right)\right|\leq c\left(1+{\left|u\right|}^{s-1}\right), \end{array}$$

for a.e. *x* ∈ *Ω*, *ω* ∈ *W*, *u* ∈ ℝ, and some *s* ∈ (1, 2_α_*), where 2_α_* = 2*n*/(*n* − *α*).

(A3) lower bound: there exist *b* ∈ ℝ and functions *γ* ∈ *L* ^2^(*Ω*, ℝ), *β* ∈ *L* ^1^(*Ω*, ℝ), such that

(16) $$\begin{array}{c} \phi \left(x,u,\omega \right)\geq -b{\left|u\right|}^{2}-\gamma \left(x\right)u-\beta \left(x\right), \end{array}$$

for a.e. *x* ∈ *Ω*, *ω* ∈ ℝ^*m*^, and *u* ∈ ℝ, where *ρ*_1_^*α*/2^ > 2*b* and *ρ*_1_ is the principal eigenvalue of the Laplace operator −Δ defined on the space *H*_0_^1^(*Ω*, ℝ).

(A4) convexity: the function *ϕ* is convex in *u*.

**Remark 2**

The principal eigenvalue *ρ*_1_ of Laplacian appears in the inequality

(17) $$\begin{array}{c} \rho_{1}^{\alpha /2}\leq \inf\left\{\frac{\int_{\Omega }^{}{\left|{\left(-\Delta \right)}^{\alpha /4}u\left(x\right)\right|}^{2}dx}{\int_{\Omega }^{}{\left|u\left(x\right)\right|}^{2}dx};\;u\in H_{0}^{\alpha /2}\left(\Omega ,\mathbb{R}\right),\;u\neq 0\right\}. \end{array}$$

Indeed, (−Δ)^*α*/4^ *u*_1_ = *ρ*_1_^*α*/4^ *u*_1_, so infimum on the right hand side of the above inequality is greater or equal to *ρ*_1_^*α*/2^. Moreover, the infimum is attained since ||*u*||_*H*_0_^*α*/2^_^2^ = ∫_*Ω*_|(−Δ)^*α*/4^ *u*(*x*)|^2^ *dx* is weakly lower semicontinuous, convex, and coercive as the norm in the reflexive space; for details, see [8, 27].

To derive the fractional Poincaré inequality of the form

(18) $$\begin{array}{c} \rho_{1}^{\alpha /2}\int_{\Omega }^{}{\left|u\left(x\right)\right|}^{2}dx\leq \int_{\Omega }^{}{\left|{\left(-\Delta \right)}^{\alpha /4}u\left(x\right)\right|}^{2}dx \end{array}$$

we apply the following theorem with *F*(*t*) = *t* ^*α*/2^.

**Theorem 3**

Let *F* be a continuous, increasing, and polynomially bounded real-valued functional on [0, *∞*), in particular, *F*(*t*) > 0 for *t* > 0. Then we have the following fractional order Poincaré inequality:

(19) $$\begin{array}{c} F\left(\sqrt{\rho_{1}}\right){||u||}_{L^{2}}\leq {||F\left(\sqrt{-\Delta }\right)u||}_{L^{2}}, \end{array}$$

compare [28, Theorem 2.8].

For the fractional Poincaré inequality with general measures involving nonlocal quantities on unbounded domain see paper by Mouhot et al. [29]. In what follows we shall also use the following result.

**Remark 4**

The fractional Sobolev inequality extending the above Poincaré inequality to *L* ^*s*^(*Ω*, ℝ) with, in general, non optimal constant *C* > 0, has the form

(20) $$\begin{array}{c} \int_{\Omega }^{}{\left|{\left(-\Delta \right)}^{\alpha /4}u\left(x\right)\right|}^{2}dx\geq C{\left(\int_{\Omega }^{}{\left|u\left(x\right)\right|}^{s}dx\right)}^{2/s} \end{array}$$

for any *s* ∈ [1, 2_α_*], *n* > *α*, and every *u* ∈ *H*_0_^*α*/2^(*Ω*, ℝ). When *s* = 2_α_* the best constant in the fractional Sobolev inequality will be denoted by *S*(*α*, *n*). This constant is explicit and independent of the domain, its exact value is

(21) $$\begin{array}{c} S\left(\alpha ,n\right)=\frac{2\pi^{\alpha /2}\Gamma \left(\left(n+\alpha \right)/2\right)\Gamma \left(\left(2-\alpha \right)/2\right){\left(\Gamma \left(n/2\right)\right)}^{\alpha /n}}{\Gamma \left(\alpha /2\right)\Gamma \left(\left(n-\alpha \right)/2\right){\left(\Gamma \left(n\right)\right)}^{\alpha /2}}, \end{array}$$

where Γ is the standard Euler Gamma function defined by Γ(*a*) = ∫_0_^*∞*^ *t* ^*a*−1^ *e* ^−*t*^ *dt*, compare [19].

When *s* = 2 we recover the fractional Poincaré inequality without an optimal constant in general.

**Remark 5**

The fractional Sobolev space *H*_0_^*α*/2^(*Ω*, ℝ) is compactly embedded into *L* ^*s*^(*Ω*, ℝ) for *s* ∈ [1, 2_α_*) and ∂*Ω* ∈ *C* ^0,1^; see [23, Corollary 7.2].

Under assumptions (A1)-(A2) the functional of action defined in (12) is well defined and Fréchet differentiable and the derivative of *F*_*ω*_ acting on *v* ∈ *H*_0_^*α*/2^(*Ω*, ℝ) has the form

(22) DFω(u)v=∫Ω[(−Δ)α/4u(x)(−Δ)α/4v(x) + ϕu(x,u(x),ω(x))v(x)]dx.

## 3. Continuous Dependence: Parameters Converging in the Strong Topology

Define {*ω*_*k*_}_*k*∈*ℕ*_ to be some sequences of parameters distributed on *Ω*. For *k* ∈ *ℕ*_0_ : = {0} ∪ *ℕ*, we denote by *U*_*k*_, the set of all possible minimizers of the functional *F*_*ω*_*k*__; that is

(23) $$\begin{array}{c} U_{k}=\left\{u\in H_{0}^{\alpha /2}\left(\Omega ,\mathbb{R}\right);F_{\omega_{k}}\left(u\right)=\underset{y\in H_{0}^{\alpha /2}\left(\Omega ,\mathbb{R}\right)}{\min}F_{\omega_{k}}\left(y\right)\right\}. \end{array}$$

Since each minimizer $\bar{u}\in U_{k}$ is a critical point of *F*_*ω*_*k*__, that is, $DF_{\omega_{k}}\left(\bar{u}\right)v=0$ for any *v* ∈ *H*_0_^*α*/2^(*Ω*, ℝ), it follows that $\bar{u}$ is a weak solution of problem (10)-(11). Inversely, if $\bar{u}$ is a weak solution of (10) satisfying (11), then $\bar{u}\in U_{k}$ provided the functional *F*_*ω*_*k*__ is convex (cf. [30, 31]). It is clear that, in general, the set *U*_*k*_ does not have to be a singleton and hence boundary value problem (10)-(11) does not have to possess a unique solution.

In the following theorem we shall use the definition of the upper Painlevé-Kuratowski limit of the sets (cf. [32]). We say that a set $\tilde{U}\subset H_{0}^{\alpha /2}\left(\Omega ,\mathbb{R}\right)$ is an upper limit of the sets *U*_*k*_, *k* ∈ *ℕ* if any point $\tilde{u}\in \tilde{U}$ is a cluster point of some sequence {*u*_*k*_}_*k*∈*ℕ*_ in *H*_0_^*α*/2^(*Ω*, ℝ) such that *u*_*k*_ ∈ *U*_*k*_ for *k* ∈ *ℕ*. By $\mathrm{limsup}U_{k}=\tilde{U}$, we shall denote the upper Painlevé-Kuratowski limit of the sets *U*_*k*_, *k* ∈ *ℕ*.

Now, we can formulate and prove the main result of this section.

**Theorem 6**

Assume that

1. the integrand *ϕ* satisfies conditions (A1)–(A3),
2. the sequence of distributed parameters {*ω*_*k*_}_*k*∈*ℕ*_ tends to *ω*_0_ in *L* ^*p*^(*Ω*, ℝ^*m*^) with *p* > 1.

Then

- for any *ω*_*k*_, the set *U*_*k*_ is a nonempty subset of *H*_0_^*α*/2^(*Ω*, ℝ), for *k* ∈ *ℕ*_0_,
- there exists a ball *B*(0, *ρ*) ⊂ *H*_0_^*α*/2^(*Ω*, ℝ) for some *ρ* > 0 such that *U*_*k*_ ⊂ *B*(0, *ρ*) for *k* ∈ *ℕ*_0_,
- any sequence {*u*_*k*_}_*k*∈*ℕ*_ such that *u*_*k*_ ∈ *U*_*k*_ is relatively compact in *H*_0_^*α*/2^(*Ω*, ℝ) and *∅* ≠ limsup⁡*U*_*k*_ ⊂ *U*_0_.

Additionally, if the sets *U*_*k*_ are singletons, that is, *U*_*k*_ = {*u*_*k*_}, *k* ∈ *ℕ*_0_, then {*u*_*k*_}_*k*∈*ℕ*_ tends to *u*_0_ in *H*_0_^*α*/2^(*Ω*, ℝ).

Before going to the proof, it is worth noting that, if *U*_*ω*_ denotes the set of all possible minimizers of the functional *F*_*ω*_ defined by (12), then assertion (c) of Theorem 6 states that the set valued mapping *L* ^*p*^(*Ω*, ℝ^*m*^)∋*ω* ↦ *U*_*ω*_ ⊂ *H*_0_^*α*/2^(*Ω*, ℝ) is upper semicontinuous with respect to the strong topology of spaces *L* ^*p*^(*Ω*, ℝ^*m*^) and *H*_0_^*α*/2^(*Ω*, ℝ).

**Proof**

Consider the following.

*Step 1.* In the first step we prove assertions (a) and (b) of our theorem.

For *k* ∈ *ℕ*_0_, consider the functional

(24) $$\begin{array}{c} F_{\omega_{k}}\left(u\right)=\int_{\Omega }^{}\left[\frac{1}{2}{\left|{\left(-\Delta \right)}^{\alpha /4}u\left(x\right)\right|}^{2}+\phi \left(x,u\left(x\right),\omega_{k}\left(x\right)\right)\right]dx.\; \end{array}$$

By assumption (2) of our theorem, ||*ω*_*k*_||_*L*^*p*^_ ≤ *C*_0_ for some *C*_0_ > 0. By (A3), we have

(25) Fωk(u)≥∫Ω[12|(−Δ)α/4u(x)|2−b|u(x)|2− γ(x)u(x)−β(x)]dx

and therefore the application of the fractional Poincaré inequality (18) gives

(26) Fωk(u)≥(12−bρ1−α/2)||u||H0α/22 −C1||u||H0α/2−C2=p(||u||H0α/2)

with *ρ*_1_^*α*/2^ − 2*b* > 0 from (A3), where *C*_1_, *C*_2_ are some constants independent of *ω*_*k*_; however, depending on ||*γ*||_*L*^2^_ and ||*β*||_*L*^1^_. The functional *F*_*ω*_*k*__ is weakly lower semicontinuous on *H*_0_^*α*/2^(*Ω*, ℝ) as a sum involving the norm in *H*_0_^*α*/2^(*Ω*, ℝ), compare [8], and the integral term with *ϕ* satisfying the standard regularity and growth conditions (A1) and (A2), compare [33–36], as *H*_0_^*α*/2^(*Ω*, ℝ) ⊂ *L* ^*s*^(*Ω*, ℝ) for suitably chosen *s* in the embedding. Since, by (26), the functionals *F*_*ω*_*k*__ are coercive, we infer that the sets *U*_*k*_ are nonempty and weakly closed. Moreover, by condition (A2), putting *u* = 0, we get the following estimates due to the boundedness of *ω*_*k*_ in *L* ^*p*^(*Ω*, ℝ^*m*^)

(27) $$\begin{array}{c} F_{\omega_{k}}\left(0\right)\leq \int_{\Omega }^{}c\left(1+{\left|\omega_{k}\left(x\right)\right|}^{p}\right)dx\leq D_{1}\;\text{if}\;1<p<\infty , \end{array}$$

(28) $$\begin{array}{c} F_{\omega_{k}}\left(0\right)\leq \int_{\Omega }^{}c\;dx\leq D_{2}\;\text{if}\;p=\infty , \end{array}$$

where the constants *D*_1_ and *D*_2_ are independent of *ω*_*k*_. Directly from inequalities (26), (27), and (28) we obtain that for some *ρ* > 0

(29) $$\begin{array}{c} U_{k}\subset B\left(0,\rho \right)=\left\{u\in H_{0}^{\alpha /2}\left(\Omega ,\mathbb{R}\right):{||u||}_{H_{0}^{\alpha /2}}\leq \rho \right\}. \end{array}$$

We have thus proved assertions (a) and (b) of our theorem.

*Step 2.* For *k* ∈ *ℕ*_0_, denote by *μ*_*k*_ the minimal value of the functional *F*_*ω*_*k*__; that is

(30) $$\begin{array}{c} \mu_{k}=\underset{u\in H_{0}^{\alpha /2}}{\min}F_{\omega_{k}}\left(u\right)=F_{\omega_{k}}\left(\bar{u}\right), \end{array}$$

where $\bar{u}\in U_{k}$. We shall observe that

(31) $$\begin{array}{c} \underset{k\to \infty }{\lim}\mu_{k}=\mu_{0} \end{array}$$

provided that *ω*_*k*_ → *ω*_0_ in *L* ^*p*^(*Ω*, ℝ^*m*^) as *k* → *∞*.

We begin by proving that the sequence {*F*_*ω*_*k*__(*u*)}_*k*∈*ℕ*_ tends to *F*_*ω*_0__(*u*) uniformly on any ball *B*(0, *ρ*) ⊂ *H*_0_^*α*/2^(*Ω*, ℝ) of radius *ρ* > 0. By definition (24), we have

(32) |Ik(u)|=|Fωk(u)−Fω0(u)|=|∫Ω[ϕ(x,u(x),ωk(x))−ϕ(x,u(x),ω0(x))]dx|.

Suppose that, on the contrary, the above integral does not tend to zero uniformly on *B*(0, *ρ*). It means that there exists *ε*_0_ > 0 and a sequence {*u*_*k*_}_*k*∈*ℕ*_ ⊂ *B*(0, *ρ*) such that |*I*_*k*_(*u*_*k*_)| > *ε*_0_. Passing to a subsequence if necessary, we can assume that {*u*_*k*_}_*k*∈*ℕ*_ tends to some $\bar{u}$ weakly in *H*_0_^*α*/2^(*Ω*, ℝ). From the fractional Sobolev compact embedding theorem, see Remark 5, we deduce that, up to subsequence, {*u*_*k*_}_*k*∈*ℕ*_ tends to $\bar{u}$ in *L* ^*s*^(*Ω*, ℝ). By assumption (2), we know that {*ω*_*k*_}_*k*∈*ℕ*_ tends to *ω*_0_ in *L* ^*p*^(*Ω*, ℝ^*m*^). Applying the Krasnoselskii theorem (cf. [37, 38]) the continuity of the operator *L* ^*s*^ × *L* ^*p*^∋(*u*, *w*) ↦ *ϕ*(·, *u*(·), *w*(·)) ∈ *L* ^1^ follows and which together with condition (A2) implies *I*_*k*_(*u*_*k*_) → 0 as *k* → *∞*. Thus we have got a contradiction with the inequality |*I*_*k*_(*u*_*k*_)| > *ε*_0_. It means that *I*_*k*_(*u*) tends to zero uniformly on *B*(0, *ρ*) and consequently {*F*_*ω*_*k*__(*u*)}_*k*∈*ℕ*_ converges to *F*_*ω*_0__(*u*) uniformly on *B*(0, *ρ*) provided that *ω*_*k*_ → *ω*_0_ in *L* ^*p*^(*Ω*, ℝ^*m*^).

Consequently, for any *ɛ* > 0 and *k* chosen to be sufficiently large, we have

(33) μk=min⁡u∈H0α/2(Ω,ℝ)⁡Fωk(u)=min⁡u∈B(0,ρ)⁡Fωk(u)≤min⁡u∈B(0,ρ)⁡Fω0(u)+ɛ=min⁡u∈H0α/2(Ω,ℝ)⁡Fω0(u)+ɛ=μ0+ɛ.

Similarly, *μ*_0_ ≤ *μ*_*k*_ + *ɛ*. We have thus proved equality (31).

*Step 3.* Finally, we shall prove assertion (c). Let {*u*_*k*_}_*k*∈*ℕ*_ be a sequence of minimizers; that is, *u*_*k*_ ∈ *U*_*k*_. Since *U*_*k*_ ⊂ *B*(0, *ρ*) for *k* ∈ *ℕ*_0_, the sequence {*u*_*k*_}_*k*∈*ℕ*_ is weakly relatively compact in *H*_0_^*α*/2^(*Ω*, ℝ). We may assume after passing to a subsequence (still denoted by *u*_*k*_) that {*u*_*k*_}_*k*∈*ℕ*_ tends to some $\bar{u}\in B\left(0,\rho \right)$ in the weak topology of *H*_0_^*α*/2^(*Ω*, ℝ). Let us prove now that $\bar{u}\in U_{0}$; that is, $\bar{u}$ is a minimizer of *F*_*ω*_0__. Indeed, suppose that $\bar{u}\notin U_{0}$. The set *U*_0_ is nonempty and therefore there exists some *u*_0_ ∈ *U*_0_ such that $u_{0}\neq \bar{u}$. Clearly, since *u*_0_ is a minimizer of *F*_*ω*_0__, $F_{\omega_{0}}\left(\bar{u}\right)-F_{\omega_{0}}\left(u_{0}\right)=\beta >0$ and moreover we have

(34) $$\begin{array}{c} \mu_{k}-\mu_{0}=\left[F_{\omega_{k}}\left(u_{k}\right)-F_{\omega_{0}}\left(u_{k}\right)\right]+\left[F_{\omega_{0}}\left(u_{k}\right)-F_{\omega_{0}}\left(\bar{u}\right)\right]+\beta . \end{array}$$

Uniform convergence of {*F*_*ω*_*k*__(*u*)}_*k*∈*ℕ*_ to *F*_*ω*_0__(*u*) on *B*(0, *ρ*) leads to *F*_*ω*_*k*__(*u*_*k*_) − *F*_*ω*_0__(*u*_*k*_) → 0 as *u*_*k*_ ∈ *B*(0, *ρ*) by (b). Furthermore, the weak lower semicontinuity of *F*_*ω*_0__ and the weak convergence of *u*_*k*_ to $\bar{u}$ in *H*_0_^*α*/2^(*Ω*, ℝ) lead to

(35) $$\begin{array}{c} \underset{k\to \infty }{\mathrm{liminf}}F_{\omega_{0}}\left(u_{k}\right)-F_{\omega_{0}}\left(\bar{u}\right)\geq 0. \end{array}$$

Thus we have got a contradiction with (31). Consequently, $\bar{u}\in U_{0}$.

What we need to do now is to demonstrate that any sequence {*u*_*k*_}_*k*∈*ℕ*_ such that *u*_*k*_ ∈ *U*_*k*_ converges strongly to $\bar{u}$ in *H*_0_^*α*/2^(*Ω*, ℝ). By (22), for *k* ∈ *ℕ*, we have

(36) $$\begin{array}{c} 0=\left(DF_{\omega_{k}}\left(u_{k}\right)-DF_{\omega_{0}}\left(\bar{u}\right)\right)\left(u_{k}-\bar{u}\right)={||u_{k}-\bar{u}||}_{H_{0}^{\alpha /2}}^{2}+I_{k}, \end{array}$$

where

(37) Ik=∫Ω(ϕu(x,uk(x),ωk(x))−ϕu(x,u¯(x),ω0(x))) ×(uk(x)−u¯(x))dx.

The Hölder inequality and the growth condition (A2) allow us to write the following estimates:

(38) Ik≤(∫Ω|ϕu(x,uk(x),ωk(x)) − ϕu(x,u¯(x),ω0(x))|s/s−1dx)(s−1)/s ×(∫Ω|uk(x)−u¯(x)|sdx)1/s≤C4(∫Ω(1+|uk(x)|s+|u¯(x)|s + |ωk(x)|p+|ω0(x)|p)dx)(s−1)/s ×||uk−u¯||Ls if  p<∞,Ik≤C5(∫Ω(1+|uk(x)|s+|u¯(x)|s)dx)(s−1)/s ×||uk−u¯||Ls if  p=∞,

where *C*_4_ and *C*_5_ are some positive constants. Since {*u*_*k*_}_*k*∈*ℕ*_ converges to $\bar{u}$ in *L* ^*s*^(*Ω*, ℝ) and {*ω*_*k*_}_*k*∈*ℕ*_ is bounded in *L* ^*p*^(*Ω*, ℝ^*m*^) we see that *I*_*k*_ → 0 as *k* → *∞* and therefore the first integral ${||u_{k}-\bar{u}||}_{H_{0}^{\alpha /2}}^{2}$ tends to zero. Thus the weak convergence of the minimizers *u*_*k*_ ∈ *U*_*k*_ to $\bar{u}\in U_{0}$ implies the strong convergence of minimizers in *H*_0_^*α*/2^(*Ω*, ℝ), which completes the proof.

Let us return to boundary value problem (10)-(11) and, for *k* ∈ *ℕ*_0_, let us denote by *S*_*k*_ the set of solutions to the problem which corresponds to the parameter *ω*_*k*_. It is the well-known fact, see, for instance, [30, 31], that for the convex functional of action the set of minimizers *U*_*k*_ coincides with the set of solutions *S*_*k*_. Hence for boundary value problem (10)-(11) we have the following corollary.

**Corollary 7**

If

1. the integrand *ϕ* satisfies conditions (A1)–(A4),
2. the sequence of distributed parameters {*ω*_*k*_}_*k*∈*ℕ*_ tends to *ω*_0_ in *L* ^*p*^(*Ω*, ℝ^*m*^) with *p* > 1,

then the sequence {*S*_*k*_}_*k*∈*ℕ*_0__ satisfies assertions (a)–(c) of Theorem 6 with *U*_*k*_ = *S*_*k*_, *k* ∈ *ℕ*_0_.

Moreover, if the functional of action is strictly convex, then for *k* ∈ *ℕ*_0_, problem (10)-(11) possesses a unique solution *u*_*k*_, and lim⁡_*k*→*∞*_ *u*_*k*_ = *u*_0_ in *H*_0_^*α*/2^(*Ω*, ℝ).

## 4. Continuous Dependence: The Parameters Converging in the Weak Topology

To achieve stronger results which are useful in optimization theory, it is necessary to narrow down the class of equations under considerations. Namely, in this section, we shall assume that the integrand *ϕ* is linear with respect to the distributed parameter *ω*; that is

(39) $$\begin{array}{c} \phi \left(x,u,\omega \right)=\phi^{1}\left(x,u\right)+\langle \phi^{2}\left(x,u\right),\omega \rangle , \end{array}$$

where *ϕ* ^1^ : *Ω* × ℝ → ℝ, *ϕ* ^2^ : *Ω* × ℝ → ℝ^*m*^, *ω* ∈ ℝ^*m*^ and 〈·, ·〉 stands for a scalar product in ℝ^*m*^. In this case, the boundary value problem (10)-(11) takes the form

(40) (−Δ)α/2u(x)+ϕu1(x,u(x))+〈ϕu2(x,u(x)),ω(x)〉=0for  x∈Ω⊂ℝn,

(41) $$\begin{array}{c} u\left(x\right)=0\;\text{for}\;x\in {\mathbb{R}}^{n}\setminus \Omega \end{array}$$

and the functional of action has the form

(42) F¯ω(u)=∫Ω[12|(−Δ)α/4u(x)|2+ϕ1(x,u(x)) +〈ϕ2(x,u(x)),ω(x)〉]dx,

where *u* ∈ *H*_0_^*α*/2^(*Ω*, ℝ) and *ω* ∈ *L* ^*p*^(*Ω*, ℝ^*m*^) with 1 < *p* < *∞*.

We impose the following conditions on *ϕ* ^1^, *ϕ* ^2^:

(A1′) regularity: the functions *ϕ* ^1^, *ϕ*_*u*_^1^, *ϕ* ^2^, and *ϕ*_*u*_^2^ are measurable with respect to *x* for any *u* ∈ ℝ and continuous with respect to *u* for a.e. *x* ∈ *Ω*;
(A2′) growth: there exists a constant *c* > 0 such that
  (43) $$\begin{array}{c} \left|\phi_{u}^{1}\left(x,u\right)\right|\leq c\left(1+{\left|u\right|}^{s-1}\right),\\ \left|\phi_{u}^{2}\left(x,u\right)\right|\leq c\left(1+{\left|u\right|}^{s-1-s/p}\right) \end{array}$$
 for a.e. *x* ∈ *Ω*, *u* ∈ ℝ and *s* ∈ (1 + 1/(*p* − 1), 2_α_*) where 2_α_* = 2*n*/(*n* − *α*) > 2 and 1 < *p* < *∞*.

Suppose that *ϕ* meets conditions (A3) and (A4). Obviously, assumptions (A1′) and (A2′) imply the function *ϕ* to satisfy (A1) and (A2). For this weaker form of the problem, the claim of the theorem on the existence and the continuous dependence can be strengthened. To draw the same conclusion this time, it suffices to assume the weak convergence of parameters.

Let {*ω*_*k*_}_*k*∈*ℕ*_ be some sequence of the distributed parameters. Denote by *U*_*k*_ a set of all minimizers of the functional of action (42) with *ω* = *ω*_*k*_ given in (23). We shall prove the following.

**Theorem 8**

Suppose that

1. the integrand *ϕ* is of the form (39) and satisfies conditions (*A*1′), (*A*2′), and (*A*3),
2. the sequence of distributed parameters {*ω*_*k*_}_*k*∈*ℕ*_ tends to *ω*_0_ in the weak topology of *L* ^*p*^(*Ω*, ℝ^*m*^).

Then the sequence {*U*_*k*_}_*k*∈*ℕ*_0__ satisfies assertions (a)–(c) of Theorem 6.

**Proof**

As in the proof of Theorem 6, in the similar manner, we obtain assertions (a) and (b) of our theorem taken from Theorem 6. Let {*u*_*k*_}_*k*∈*ℕ*_ ⊂ *H*_0_^*α*/2^(*Ω*, ℝ) be an arbitrary sequence such that *u*_*k*_ ∈ *U*_*k*_ ⊂ *B*(0, *ρ*), for *k* ∈ *ℕ*, where the sets *U*_*k*_ are defined by formula (23). The sequence {*u*_*k*_}_*k*∈*ℕ*_ is bounded and therefore weakly relatively compact. Passing, if necessary, to a subsequence, we can assume that *u*_*k*_⇀*u*_0_ weakly in *H*_0_^*α*/2^(*Ω*, ℝ). We shall show that *u*_0_ ∈ *U*_0_, but now we present different approach than in the proof of Theorem 6. By conditions (A1′) and (A2′) and formula (22), for *k* ∈ *ℕ* and *h* ∈ *H*_0_^*α*/2^(*Ω*, ℝ), we have

(44) 0=DF¯ωk(uk)h=∫Ω[(−Δ)α/4uk(x)(−Δ)α/4h(x)   +ϕu1(x,uk(x))h(x) +〈ϕu2(x,uk(x))h(x),ωk(x)〉]dx.

It is easy to observe that since *u*_*k*_⇀*u*_0_ weakly in *H*_0_^*α*/2^(*Ω*, ℝ) for any *h* ∈ *H*_0_^*α*/2^(*Ω*, ℝ)

(45) lim⁡k→∞∫Ω(−Δ)α/4uk(x)(−Δ)α/4h(x)dx   =∫Ω(−Δ)α/4u0(x)(−Δ)α/4h(x)dx.

By the fractional Sobolev compact embedding theorem, after passing to a subsequence (still denoted by *u*_*k*_) if necessary, we can assume that {*u*_*k*_}_*k*∈*ℕ*_ tends to *u*_0_ in *L* ^*s*^(*Ω*, ℝ) for *s* ∈ (1, 2_α_*). By (A2′), the superposition operator *ϕ*_*u*_^1^(·, *u*(·))*h*(·) acting on *u* ∈ *L* ^*s*^(*Ω*, ℝ) to *L* ^1^(*Ω*, ℝ) is continuous; that is, for any *h* ∈ *H*_0_^*α*/2^(*Ω*, ℝ)

(46) $$\begin{array}{c} \underset{k\to \infty }{\lim}\int_{\Omega }^{}\phi_{u}^{1}\left(x,u_{k}\left(x\right)\right)h\left(x\right)dx=\int_{\Omega }^{}\phi_{u}^{1}\left(x,u_{0}\left(x\right)\right)h\left(x\right)dx. \end{array}$$

Let us consider the integral

(47) $$\begin{array}{c} I_{k}=\int_{\Omega }^{}\langle \phi_{u}^{2}\left(x,u_{k}\left(x\right)\right)h\left(x\right),\omega_{k}\left(x\right)\rangle dx \end{array}$$

which can be represented as

(48) $$\begin{array}{c} I_{k}=I_{k}^{1}+I_{k}^{2}, \end{array}$$

where

(49) Ik1=∫Ω〈ϕu2(x,u0(x))h(x),ωk(x)〉dx,Ik2=∫Ω〈[ϕu2(x,uk(x))h(x) − ϕu2(x,u0(x))h(x)],ωk(x)〉dx.

Since ||*ω*_*k*_||_*L*^*p*^_||*h*||_*L*^*s*^_ ≤ *C*, we see that

(50) $$\begin{array}{c} \left|I_{k}^{2}\right|\leq C{\left(\int_{\Omega }^{}{\left|\phi_{u}^{2}\left(x,u_{k}\left(x\right)\right)-\phi_{u}^{2}\left(x,u_{0}\left(x\right)\right)\right|}^{\gamma }dx\right)}^{1/\gamma }, \end{array}$$

where *γ* = *ps*/((*s* − 1)*p* − *s*) and moreover due to growth estimate (A2′) we get the bound *C*(1 + ||*u*_*k*_||_*L*^*s*^_ + ||*u*_0_||_*L*^*s*^_), since

(51) $$\begin{array}{c} \frac{ps}{\left(s-1\right)p-s}\frac{\left(s-1\right)p-s}{p}=s. \end{array}$$

Hence, up to subsequence, we have that *I*_*k*_^2^ → 0 as *k* → *∞*. Similarly, by (2) and (A2′) we get

(52) $$\begin{array}{c} \underset{k\to \infty }{\lim}I_{k}^{1}=\int_{\Omega }^{}\langle \phi_{u}^{2}\left(x,u_{0}\left(x\right)\right)h\left(x\right),\omega_{0}\left(x\right)\rangle dx. \end{array}$$

Thus

(53) $$\begin{array}{c} \underset{k\to \infty }{\lim}I_{k}=\int_{\Omega }^{}\langle \phi_{u}^{2}\left(x,u_{0}\left(x\right)\right)h\left(x\right),\omega_{0}\left(x\right)\rangle dx \end{array}$$

for all *h* ∈ *H*_0_^*α*/2^(*Ω*, ℝ). Taking into account equalities (45), (46), and (53), we infer that $D{\bar{F}}_{\omega_{0}}\left(u_{0}\right)=0$. It means that *u*_0_ ∈ *U*_0_. To complete the proof, we shall verify that the sequence {*u*_*k*_}_*k*∈*ℕ*_ converges to *u*_0_ in *H*_0_^*α*/2^(*Ω*, ℝ). By (22), we have

(54) 0=(DF¯ωk(uk)−DF¯ω0(u0))(uk−u0)=∫Ω|(−Δ)α/4uk(x)−(−Δ)α/4u0(x)|2dx +∫Ω(ϕu1(x,uk(x))−ϕu1(x,u0(x)))(uk(x)−u0(x))dx +∫Ω〈ϕu2(x,uk(x))(uk(x)−u0(x)),ωk(x)〉dx −∫Ω〈ϕu2(x,u0(x))(uk(x)−u0(x)),ω0(x)〉dx=||uk−u0||H0α/22+Ik1+Ik2+Ik3.

Since for

(55) $$\begin{array}{c} I_{k}^{1}=\int_{\Omega }^{}\left(\phi_{u}^{1}\left(x,u_{k}\left(x\right)\right)-\phi_{u}^{1}\left(x,u_{0}\left(x\right)\right)\right)\left(u_{k}\left(x\right)-u_{0}\left(x\right)\right)dx, \end{array}$$

by the Hölder inequality and the growth condition (A2′), we get

(56) Ik1≤(∫Ω|ϕu1(x,uk(x))−ϕu1(x,u0(x))|s/(s−1)dx)(s−1)/s ×(∫Ω|uk(x)−u0(x)|sdx)1/s≤C4(∫Ω(1+|uk(x)|s+|u0(x)|s)dx)(s−1)/s ×||uk−u0||Ls if  p<∞,

and in a similar manner using the Hölder inequality, *I*_*k*_^2^ and *I*_*k*_^3^ can be estimated by the terms involving ||*u*_*k*_−*u*_0_||_*L*^*s*^_, ||*w*_*k*_||_*L*^*p*^_, for *k* ∈ *ℕ*_0_, and finally ||*ϕ*_*u*_^2^||_*L*^*ps*/(*p*(*s*−1)−*s*)^_. The latter term due to the growth condition imposed on *ϕ*_*u*_^2^ can be estimated as before from the above by ||*u*_*k*_||_*L*^*s*^_ for *k* ∈ *ℕ*_0_. Since {*u*_*k*_}_*k*∈*ℕ*_ converges to *u*_0_ in *L* ^*s*^(*Ω*, ℝ) and {*ω*_*k*_}_*k*∈*ℕ*_ converges to *ω*_0_ in *L* ^*p*^(*Ω*, ℝ^*m*^) we have *I*_*k*_ = (*I*_*k*_^1^ + *I*_*k*_^2^ + *I*_*k*_^3^) → 0 as *k* → *∞*. Consequently, ||*u*_*k*_−*u*_0_||_*H*_0_^*α*/2^_ → 0 as *k* → 0. Thus, the weak convergence of minimizers *u*_*k*_ ∈ *U*_*k*_ to *u*_0_ ∈ *U*_0_ implies the strong convergence of minimizers in *H*_0_^*α*/2^(*Ω*, ℝ). Therefore, the proof of our theorem is complete.

## 5. Existence of Optimal Solutions

We now formulate the optimal control problem to which this section is dedicated. It transpires that the continuous dependence results from Section 4 enable us to prove a theorem on the existence of optimal processes to some optimal control problem. Specifically, we shall consider control problem governed by boundary value problem (40)-(41) with the integral cost functional

(57) $$\begin{array}{c} J\left(u,\omega \right)=\int_{\Omega }^{}\theta \left(x,u\left(x\right),{\left(-\Delta \right)}^{\alpha /4}u\left(x\right),\omega \left(x\right)\right)dx, \end{array}$$

where *θ* : *Ω* × ℝ × ℝ × ℝ^*m*^ → ℝ is a given function. Here *u* ∈ *H*_0_^*α*/2^(*Ω*, ℝ) is the trajectory and *ω* ∈ *𝒲* is the distributed control where

(58) $$\begin{array}{c} 𝒲=\left\{\omega \in L^{p}\left(\Omega ,{\mathbb{R}}^{m}\right):\omega \left(x\right)\in W\;\;\text{for}\;\text{a}.\text{e}.\;x\in \Omega \right\} \end{array}$$

with *p* > 1 and *W* being a compact and convex subset of ℝ^*m*^.

Let *𝒟* be the set of all admissible pairs; that is

(59) 𝒟={(u,ω)∈H0α/2(Ω,ℝ) × 𝒲:u  satisfies  (40)  for  ω∈𝒲}.

It should be noted that under assumptions of Theorem 8 the set of all admissible pairs *𝒟* is nonempty. In this section, our aim is to find a pair (*u*_ω*_, ω*) ∈ *H*_0_^*α*/2^(*Ω*, ℝ) × *𝒲* such that

(60) $$\begin{array}{c} J\left(u_{\omega^{*}},\omega^{*}\right)=\underset{\left(u,\omega \right)\in 𝒟}{\min}J\left(u,\omega \right). \end{array}$$

On the integrand *θ* we impose the following conditions.

(A5) The function *θ* = *θ*(*x*, *u*, *p*, *ω*) is measurable with respect to *x* for all *u* ∈ ℝ, *p* ∈ ℝ, *ω* ∈ *W*, continuous with respect to (*u*, *p*, *ω*) for a.e. *x* ∈ *Ω*, and convex with respect to *ω* for all *u* ∈ ℝ, *p* ∈ ℝ, and a.e. *x* ∈ *Ω*. Moreover there exists a constant *c* > 0 such that
  (61) $$\begin{array}{c} \left|\theta \left(x,u,p,\omega \right)\right|\leq c\left(1+{\left|u\right|}^{s}+{\left|p\right|}^{2}\right) \end{array}$$
 for a.e. *x* ∈ *Ω*, all *u* ∈ ℝ, *p* ∈ ℝ, *ω* ∈ *W*, and for some *s* ∈ (1, 2_α_*) where 2_α_* = 2*n*/(*n* − *α*).
(A6) There exists a function *η* ∈ *L* ^1^(*Ω*, ℝ) and a constant *M* > 0 such that
  (62) $$\begin{array}{c} \theta \left(x,u,p,\omega \right)\geq \eta \left(x\right)-M\left(\left|u\right|+\left|p\right|+\left|\omega \right|\right) \end{array}$$
 for all *u* ∈ ℝ, *p* ∈ ℝ, *ω* ∈ *W*, and a.e. *x* ∈ *Ω*.

Now we prove a theorem on the existence of optimal processes to our optimal control problem (60).

**Theorem 9**

If the functions *ϕ* of the form (39) satisfies (*A*1′), (*A*2′), (A3), (A4), and the integrand *θ* meets assumptions (A5), (A6), then the optimal control problem (60) possesses at least one optimal process (*u*_ω*_, ω*).

**Proof**

From (A5), (A6), and classical theorems on semicontinuity of integral functional (cf. [33–36]), we deduce that *J* is lower semicontinuous with respect to the strong topology in the space *H*_0_^*α*/2^(*Ω*, ℝ) and the weak topology of *L* ^*p*^(*Ω*, ℝ^*m*^), since convergence of any sequence {*u*_*k*_}_*k*∈*ℕ*_ in *H*_0_^*α*/2^(*Ω*, ℝ) implies the strong convergence of {*u*_*k*_}_*k*∈*ℕ*_ in *L* ^*s*^(*Ω*, ℝ) with *s* ∈ (1, 2_α_*) and the strong convergence of {(−Δ)^*α*/4^ *u*_*k*_}_*k*∈*ℕ*_ in *L* ^2^(*Ω*, ℝ).

Let {(*u*_*k*_,*ω*_*k*_)}_*k*∈*ℕ*_ ⊂ *𝒟* be a minimizing sequence for optimal control problem (60); that is

(63) $$\begin{array}{c} \underset{k\to \infty }{\lim}J\left(u_{k},\omega_{k}\right)=\underset{\left(u,\omega \right)\in 𝒟}{\inf}J\left(u,\omega \right)=\vartheta , \end{array}$$

Since the set *W* is compact and convex, the sequence {*ω*_*k*_}_*k*∈*ℕ*_ is compact in the weak topology of *L* ^*p*^(*Ω*, ℝ^*m*^). Passing to subsequence, if necessary, we can assume that *ω*_*k*_ tends to some *ω*_0_ ∈ *𝒲* weakly in *L* ^*p*^(*Ω*, ℝ^*m*^). By assumption (A4) the set of the weak solutions of problem (40)-(41) coincides with the set of minimizers of the functional ${\overset{-}{F}}_{\omega }$ on the space *H*_0_^*α*/2^(*Ω*, ℝ). By Theorem 8, the sequence {*u*_*k*_}_*k*∈*ℕ*_, or at least some of its subsequence, tends to *u*_0_ in *H*_0_^*α*/2^(*Ω*, ℝ) and the pair (*u*_0_, *ω*_0_) is an admissible pair for control problem (40)-(41).

Due to the lower semicontinuity of *J*, we have

(64) $$\begin{array}{c} J\left(u_{0},\omega_{0}\right)\leq \underset{k\to \infty }{\mathrm{liminf}}J\left(u_{k},\omega_{k}\right) \end{array}$$

provided *u*_*k*_ tends to *u*_0_ in *H*_0_^*α*/2^(*Ω*, ℝ) and *ω*_*k*_⇀*ω*_0_ weakly in *L* ^*p*^(*Ω*, ℝ^*m*^). Furthermore, by (63) and (64), we have

(65) $$\begin{array}{c} \vartheta \leq J\left(u_{0},\omega_{0}\right)\leq \underset{k\to \infty }{\mathrm{liminf}}J\left(u_{k},\omega_{k}\right)=\underset{\left(u,\omega \right)\in 𝒟}{\inf}J\left(u,\omega \right)=\vartheta . \end{array}$$

Thus, *J*(*u*_0_, *ω*_0_) = *ϑ* = inf⁡_(*u*,*ω*)∈*𝒟*_ *J*(*u*, *ω*). It means that the process (*u*_ω*_, ω*) = (*u*_0_, *ω*_0_) is optimal for the problem (60).

**Remark 10**

From the proof of Theorem 9 one can see that it suffices to assume weaker assumption on controls than *W* to be compact and convex, namely only boundedness of *ω*_*k*_ in *L* ^*p*^(*Ω*, ℝ^*m*^).

**Remark 11**

By a direct calculation, one can check that the quadratic functional

(66) $$\begin{array}{c} ℱ\left(u\right)=\frac{1}{2}\int_{\Omega }^{}\left({\left|{\left(-\Delta \right)}^{\alpha /4}u\left(x\right)\right|}^{2}-\xi {\left|u\left(x\right)\right|}^{2}\right)dx \end{array}$$

is strictly convex for *ξ* < *ρ*_1_^*α*/2^ and convex for *ξ* = *ρ*_1_^*α*/2^ where *ρ*_1_ is the principal eigenvalue of the operator −Δ defined on *H*_0_^1^(*Ω*, ℝ).

Since

(67) F¯ω(u)=ℱ(u) +∫Ω(ξ2|u(x)|2+ϕ1(x,u(x))  +〈ϕ2(x,u(x)),ω(x)〉)dx

*u* ∈ *H*_0_^*α*/2^(*Ω*, ℝ). Theorem 9 implies the following.

**Corollary 12**

The optimal control system (60) possesses at least one optimal process (*u*_ω*_, ω*) provided the functions *ϕ* of the form (39) satisfy (*A*1′), (*A*2′), and (*A*3), the integrand *θ* meets assumptions (A5) and (A6) and the function (*ξ*/2)|*u*|^2^ + *ϕ* ^1^(*x*, *u*) + 〈*ϕ* ^2^(*x*, *u*), *ω*〉 is convex in *u* for some *ξ* ≤ *ρ*_1_^*α*/2^, all *ω* ∈ *W* and a.e. *x* ∈ *Ω*.

**Example 13**

Let *Ω* be a cube of the form

(68) $$\begin{array}{c} \Omega =P^{3}\left(0,\pi \right)=\left\{x\in {\mathbb{R}}^{3}:0<x^{i}<\pi ,\;i=1,2,3\right\}. \end{array}$$

Note that *u*_1_ = sin*x*_1_sin*x*_2_sin*x*_3_ and *ρ*_1_ = 3 are eigenfunction and eigenvalue for −Δ on *H*_0_^1^(*Ω*, ℝ) since −Δ*u*_1_ = 3*u*_1_. Similarly, (−Δ)^*α*/2^ *u*_1_ = 3^*α*/2^ *u*_1_ hence, by (4), 3^*α*/2^ is the first eigenvalue for (−Δ)^*α*/2^ in this case. The equation is of the form

(69) (−Δ)α/2u(x)−au(x)+s|x|2us−1(x)ω1(x)−|x|ω2(x)=0for  x∈P3(0,π),u(x)=0 on⁡  ℝ3∖P3(0,π)

for 1 < *s* < 6/(3 − *α*), 1 < *p* sufficiently large and the cost is given by

(70) J(u,ω)=∫Ω[us(x)+|(−Δ)α/4u(x)|2ω1(x)  − |x|(−Δ)α/4u(x)+|ω(x)|2−ω2(x)]dx,

where *a* < 3^*α*/2^, 0 ≤ *ω* ^1^(*x*) ≤ 1, and 0 ≤ *ω* ^2^(*x*) ≤ 1. Obviously, the functional of action for system (69) has the form

(71) F¯ω(u)=∫Ω[12|(−Δ)α/4u(x)|2−a2u2(x) +|x|2us(x)ω1(x)−u(x)|x|ω2(x)]dx.

It is easy to check that the functionals ${\bar{F}}_{\omega }$ and *J* satisfy all assumptions of Theorems 8 and 9. By Remark 11, ${\bar{F}}_{\omega }$ is strictly convex. Thus, Theorem 9 implies that for any control *ω* there exists exactly one solution *u*_*ω*_ of (69) and the solution continuously depends on control *ω*. Moreover, by Corollary 12, we infer that there exists optimal control (*u*_ω*_, ω*) described by (69) with the cost functional given by (70).

## 6. Summary

In this paper we formulate some sufficient condition under which the boundary value problem considered in the paper possesses at least one solution which continuously depends on distributed parameters. We based our approach on the variational methods and we have investigated the stability problem or continuous dependence problem for the problem involving fractional Laplace operator in the fractional Sobolev space *H*_0_^*α*/2^(*Ω*, ℝ) with distributed parameters *ω* from the space *L* ^*p*^(*Ω*, ℝ^*m*^) thus generalizing the stability results obtained for the boundary value problem with the Laplace operator in [1–3]. The stability results enable us to prove the theorem on the existence of optimal processes to some control problem with the integral cost functional.

The question of the existence of a solution for the boundary value problem of the Dirichlet type, periodic, homoclinic or heteroclinic type, and so forth was investigated in many papers and monographs. One can find a wide survey of results and research methods in monographs [30, 31, 38–41] and the references to be found therein. On the contrary to the initial value problem the literature on the stability problems for the boundary value problems governed by the differential equation of the elliptic type is not very vast. The stability of solutions of scalar second-order ordinary differential equation with two-point boundary conditions based on some direct methods related to the implicit function theorem was considered among others in the papers [42–46].

The question of the continuous dependence of solutions of the linear elliptic equations with the variable Dirichlet boundary data and parameters was investigated in the pioneering paper of Oleĭnik compare [47]. In this work sufficient conditions for stability of the linear partial differential equation defined in the classical spaces of smooth solutions were formulated. Analogous results for the scalar linear partial differential equation with the Dirichlet boundary conditions defined on the Sobolev spaces were proved in the paper [48]. The results on the stability of multidimensional nonlinear boundary value problems with variable parameters appeared in papers [49–51] where ordinary differential equations with two-point boundary conditions and variable functional parameters were investigated, and the stability conditions with respect to the strong and weak topology were proved. Similar results for partial differential equation with distributed parameters are given in papers [1–3, 52, 53].
